# Nicotinic Acetylcholine Receptor Pathways in Cancer: From Psychiatric Clues to Therapeutic Opportunities

**DOI:** 10.1002/cnr2.70387

**Published:** 2025-11-11

**Authors:** Mohammad Hossein Azadi, Pouya Pazooki, Soheila Ajdary, Hamed Shafaroodi

**Affiliations:** ^1^ Faculty of Cellular and Molecular Sciences College of Biological Sciences, Kharazmi University Tehran Iran; ^2^ Cumming School of Medicine University of Calgary Calgary Alberta Canada; ^3^ Department of Immunology Pasteur Institute of Iran Tehran Iran; ^4^ Pharmacology Department, School of Medicine Tehran University of Medical Sciences Tehran Iran

**Keywords:** breast cancer, lung cancer, molecular oncology, nAChRs, neurotransmitters, prostate cancer, signal transduction

## Abstract

**Background:**

The prevalence of cancer poses significant challenges to treatment, largely because of drug resistance along with other side effects. Current studies have been investigating the growth factors more than other biologic tumor features, such as neurobiologic features. Here in this review, we highlight the role of nicotinic acetylcholine receptors (nAChRs) in cancer development with their context‐dependent activation and downstream effectors.

**Recent Findings:**

Some nAChR subtypes stimulate tumorigenic pathways, EGFR/ERK1/2, PI3K/AKT, and MAPK, with varying responses based on the receptor subtype and tissue type. Notably, the Src kinase and MAPK pathways are common downstream effectors in lung, breast, and prostate cancers despite the variations in the predominant nAChR subunits in each cancer: α7 in lung, α9 in breast, and likely α5 and α7 in prostate tumors.

**Conclusion:**

These findings underscore the importance of targeting nAChRs in a context‐specific manner to modulate shared signaling pathways, particularly the acetylcholine‐stimulated Src/MAPK pathway. This review also calls for more investigation on other neurotransmitters and potential common pathways, as implicated by psychological reports, to advance the understanding of cancer biology and therapies.

## 1 Introduction

The rising prevalence of cancer presents formidable challenges in treatment, including drug resistance and adverse side effects. Current research predominantly emphasizes growth factors that have received a greater number of reported approvals from the Food and Drug Administration (FDA) [1, 2] while overlooking other critical aspects of tumor biology. Exploring tumors through neuroscientific and psychiatric lenses unveils intriguing perspectives. The claim that modern studies predominantly focus on growth factors at the expense of other critical aspects of tumor biology raises concerns about the lack of investigation of neurobiological, psychological, and neuroendocrine processes involved in tumorigenesis [3, 4]. For instance, psychological factors such as depression and stress impact tumor development through neuroendocrine routes, including catecholamine discharge initiated through sympathetic nervous system stimulation, enhancing angiogenesis and immune evasion [5, 6]. In addition, neuroimmune communications play a critical role in cytokine (e.g., NF‐κB, TNF‐α) discharge in the microenvironment of a tumor, impacting immune cell function and immune evasion [7, 8]. These examples illustrate the imperative for extending study frameworks beyond growth factors to include neural, endocrine, and psychological dimensions, potentially revealing new therapeutic targets and enhancing our understanding of tumor biology.

Over recent decades, investigations into the interplay between mood and cancer risk have yielded conflicting findings [9, 10]. Some studies have highlighted a robust correlation between emotional states and mortality rates among cancer patients [11]. Notably, research has demonstrated varying susceptibility to different cancer types based on individual personality traits [12]. Additionally, emerging evidence underscores the significant impact of motivation on reducing cancer susceptibility and enhancing treatment efficacy [13, 14]. On the other hand, even just mild symptoms of depression can steeply amplify cancer mortality rates [10]. Diagnostic assessments of depression in cancer patients often rely on the criteria outlined in the Diagnostic and Statistical Manual of Mental Disorders (DSM) [15, 16]. These criteria assess the depressive symptoms in the individual for at least 14 days, such as an increase or decrease in weight, sleep disturbances, differences in motor function, unrelenting fatigue, feelings of worthlessness or guilt, difficulties in concentration, and recurrent thoughts of death and suicide unrelated to external factors [17, 18]. Moreover, the World Health Organization (WHO) recognizes psychological factors as risk factors for breast and lung cancer development [19]. Studies also highlight mood's influence on the immune system, evidenced by altered expression of inflammatory mediators like NFκB during depressive states [20], leading to compromised immune responses against cancer [21]. Chronic stress further impacts genetic pathways, potentially promoting tumor growth [6].

The intricate nervous system network underpins these emotional responses, with neurotransmitters and hormones playing pivotal roles in shaping behaviors and emotions [22, 23]. Notably, dysregulation of acetylcholine has indeed depicted associations with depression, with acetylcholine receptor antagonists emerging as promising candidates for rapid antidepressant treatment [9]. Acetylcholine receptor pathways interact with various neurotransmitter systems like serotonin, dopamine, and norepinephrine [24, 25], modulating these potential pathways as a therapeutic classifier in cancer patients. Understanding the biological groundings of emotions and their impact on tumors has always been complex issues and tell us about very nuanced interactions in neural and peripheral systems. This study endeavors to integrate psychological and biological perspectives for further considerations of potential tumor signaling pathways. This will be one of the earliest to look into the related pathways signaling with nicotinic acetylcholine receptors (nAChRs) through the scope of psychiatric oncology toward devising novel cancer treatment methodologies.

All studies relevant to the review topic were found through comprehensive searches using PubMed, Google Scholar, Scopus, and Embase databases. The search strategy combined several medical subject headings (MeSH) terms: “Nicotinic Acetylcholine Receptors,” “Breast Cancer,” “Lung Cancer,” “Prostate Cancer,” and “Signal Transduction,” along with other words drawn from the free text of the literature. The final search query tried to be as encompassing as possible of all relevant studies published in English. These citations arise out of works written within the recent decade; thus, they provide recent and wide consideration of the literature available as regards acetylcholine signaling in cancer development. The selected studies had to investigate either molecular mechanisms, signaling pathways, expression patterns of receptors, or any therapeutic implications of nAChRs in cancer biology. Both in vitro and in vivo models were included to ensure a broad evaluation of experimental evidence across different research contexts.

Results were grouped based on distinct cancer types (breast, lung, prostate) and specific nAChR subtypes (e.g., α7, α9, α5), aligning them with their relevant key signaling pathways (e.g., EGFR/ERK1/2, PI3K/AKT, MAPK), and summarizing their contributions to cancer progression. The comparison is hence done on the collected organized data of each cell line and subtype to find key pathways in common. In addition, the possibility of targeting nAChRs in the cancer treatment arena was discussed.

## 2 The Connection Between Central Acetylcholine Levels in Psychiatric Disorders and Peripheral Acetylcholine Secretion in Tumors

The relationship between psychiatric disorders and tumors deals with a supercomplex interfacing with the neurotransmitter system of the central nervous system (CNS) and its effects on the peripheral nervous system (PNS) particularly in the tumor microenvironment (TME) [26, 27]. Disorders of neurotransmission in the CNS have a great amount of reprisal in the symptoms of mental illnesses, which, in turn, can confer metastatic and invasive proneness to invasion through the release of peripheral neurotransmitters in TME [27]. This raises questions about the correlation between fluctuations in brain acetylcholine levels during psychiatric disorders and its peripheral release.

Studies have highlighted the CNS–PNS connection facilitated by specialized transporters across the blood–brain barrier (BBB) [28, 29]. Notably, peripheral neurotransmitters, including acetylcholine, provide insights into CNS functioning, as evidenced by their presence in glandular cells like adrenal, enterochromaffin cells, and mast cells [30, 31]. Despite ambiguities in directly correlating PNS and CNS, clinical assessments show promise in the existence of the links between these two arms of the nervous system [32]. Within the TME, tumors demonstrate diverse origins of acetylcholine production. Gastrointestinal tumors exhibit autocrine acetylcholine secretion, with the vagus nerve and tuft cells identified as primary sources [33]. Similarly, lung cancer cells express acetylcholine through autocrine growth factor pathways involving nicotinic and muscarinic receptors [34]. Researchers underscored a critical role played by nicotinic acetylcholine receptors in the stress‐induced release of catecholamines from points of the adrenal medulla and sympathetic nerve endings, thus possibly enhancing the effects of smoking and stress on cancer progress [31, 35].

## 3 Acetylcholine Receptors Structure

Acetylcholine as a crucial neurotransmitter is synthesized in cholinergic neurons through the enzymatic combination of acetyl‐CoA and choline [36]. This key neurotransmitter, besides extremely varied neuronal information, acts as an autocrine hormone produced by many non‐neuronal cells, such as those originating from the intestines, airways, and immune system [7, 37, 38]. The idea that cholinergic receptors spread out all over the body further explains the relationship between nicotine exposure and cancer promotion.

**FIGURE 1:**
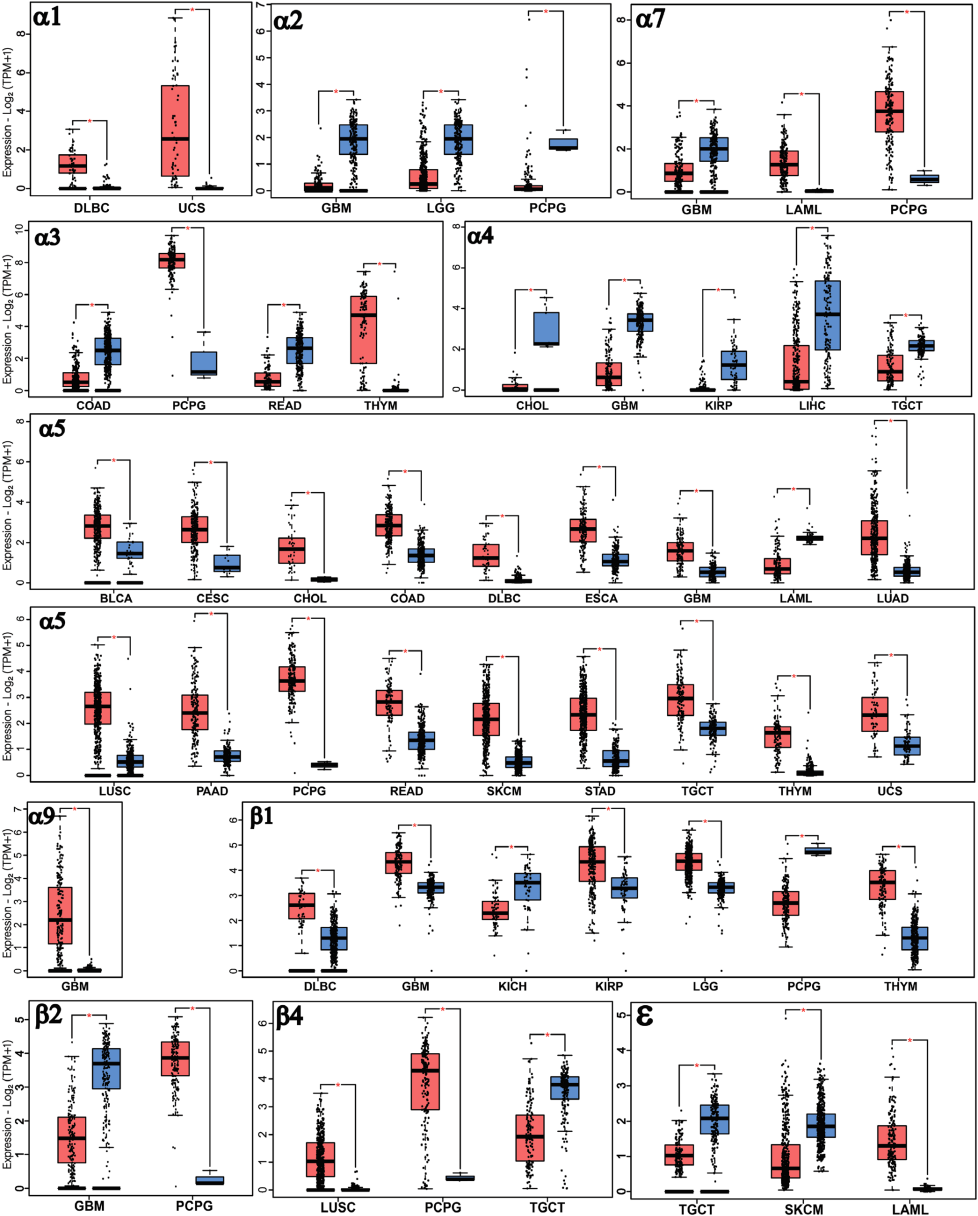
Comparison of the expression of different subunits of nicotinic acetylcholine receptors in cancerous and normal cells (based on TCGA and GTEx datasets). The red color indicates the expression level of the receptor subunit in cancerous cells, while blue represents the expression in normal cells. This chart only reports statistically significant differences (*p* < 0.05) in subunit expression between normal and cancerous cells. For this reason, subunits α6, α10, β3, γ, and ε are not displayed due to the lack of significant differences between cancerous and normal samples. Data extracted and analyzed from Gepia2.cancer‐pku.cn. BLCA, bladder urothelial carcinoma; BRCA, breast invasive carcinoma; CESC, cervical squamous cell carcinoma and endocervical adenocarcinoma; CHOL, cholangiocarcinoma; COAD, colon adenocarcinoma; DLBC lymphoid neoplasm diffuse large B‐cell lymphoma; ESCA, esophageal carcinoma; GBM, glioblastoma multiforme; HNSC, head and neck squamous cell carcinoma; KICH, kidney chromophobe; KIRP, kidney renal papillary cell carcinoma; LAML, acute myeloid leukemia; LGG, brain lower grade glioma; LIHC, liver hepatocellular carcinoma; LUAD, lung adenocarcinoma; LUSC, lung squamous cell carcinoma; PAAD, pancreatic adenocarcinoma; PCPG, pheochromocytoma and paraganglioma; PRAD, prostate adenocarcinoma; READ, rectum adenocarcinoma; SKCM, skin cutaneous melanoma; STAD, stomach adenocarcinoma; TGCT, testicular germ cell tumors; THCA, thyroid carcinoma; THYM, thymoma; UCS, uterine carcinosarcoma.

There are two types of acetylcholine receptors; the first subtype is the muscarinic acetylcholine receptor (mAChR) stimulated by muscarine while the other subtype, nAChR is stimulated by nicotine. Muscarinic receptors have five subtypes (M1 to M5) and are G protein‐coupled receptors (GPCRs). Nicotinic receptors are ion channels formed of five transmembrane subunits (mammals have seventeen subunits) [39, 40, 41]. Nicotinic receptors are classified into neuronal (Nn or N2 type) and muscle (Nm or N1 type). Neuronal nicotinic receptors include homopentamers (e.g., α7, α8, α9) and heteropentamers (e.g., α2‐α9 combined with β2‐β4 or α9 and α10), which are distributed throughout both central and peripheral nervous systems. The muscle nicotinic receptors, formed by the α1, β1, and δ subunits and additionally either the γ subunit (in fetuses) or ε subunit (in adults), are most commonly present at the neuromuscular junction [42, 43]. The expression of different subunits of nAChRs was examined through data in the gene expression profiling interactive analysis (GEPIA) web server, as indicated in Figure 1. The research worked using data obtained from the cancer genome atlas (TCGA) and genotype‐tissue expression (GTEx) databases [4, 44].

Since nicotinic receptors can form heteromeric complexes, the expression of nAChRs subunits does not necessarily correlate with functional receptor levels. In this study, while tumor profiling primarily focuses on acetylcholine receptor expression, the relative abundance of subunit expression is critically important. Furthermore, the synergistic interactions between these subunits should not be overlooked.

**FIGURE 2:**
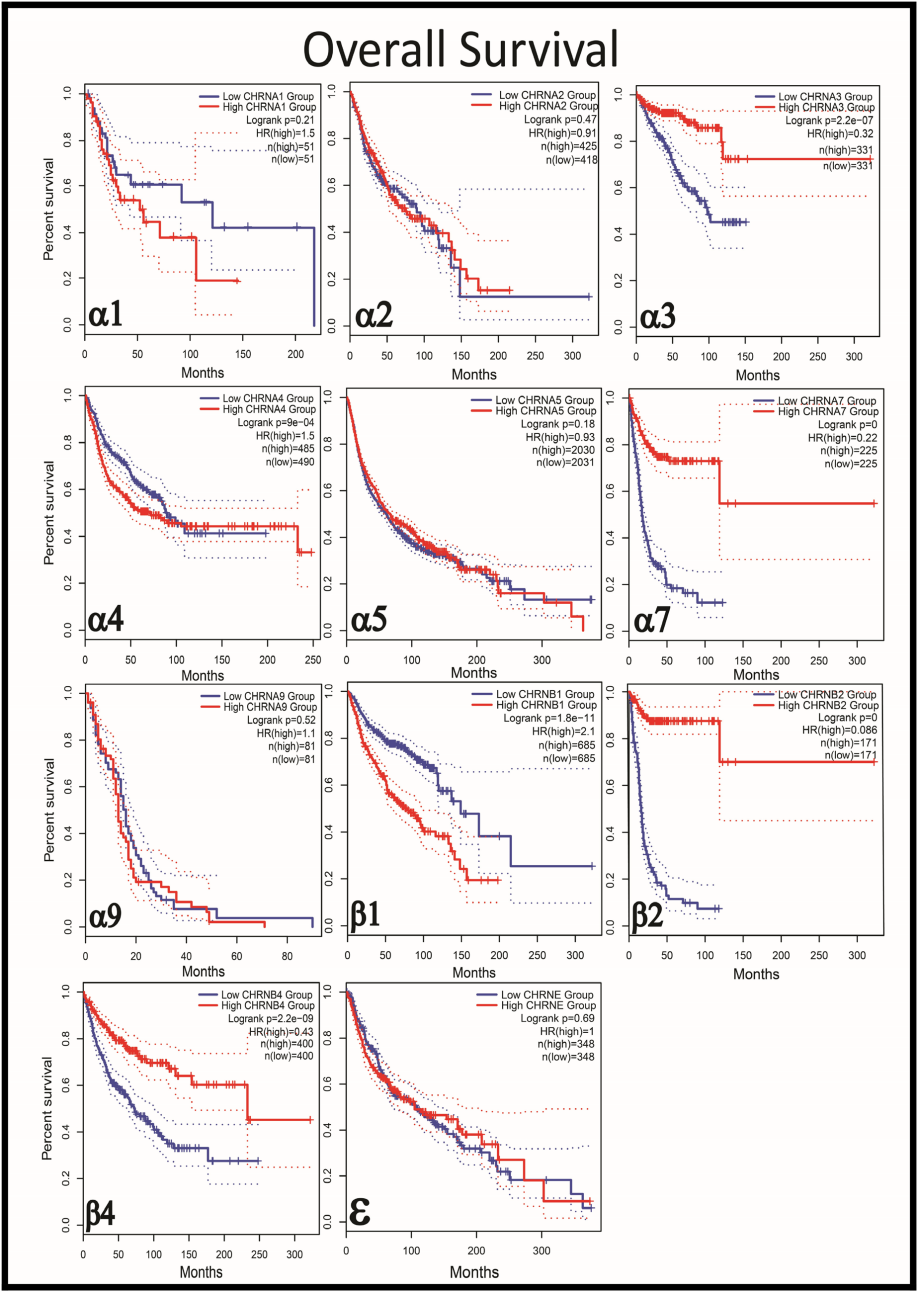
The Kaplan–Meier survival curves comparing overall survival between patients with high expression (red line) and low expression (blue line) of nAChR subtypes. For each subtype, only data from specific cancers (listed in Figure 1) with significantly high receptor expression (*p* < 0.05) were analyzed. The divergence in survival curves suggests that differential expression of nAChR subtypes may influence clinical outcomes, with high expression of certain subtypes correlating with either improved or reduced survival, depending on the cancer type. Data extracted and analyzed from Gepia2.cancer‐pku.cn.

As illustrated in Figure 2, overall survival (OS) was exclusively analyzed in tumors exhibiting statistically significant nAChR expression (Figure 1). Notably, a substantial survival difference was observed only in specific cancer types, underscoring the critical notion that nAChR‐mediated therapeutic strategies are not universally applicable across all malignancies. This striking variability in patient outcomes reinforces the context‐dependent biological roles of nAChR subtypes, which appear to be functionally relevant only in specific tumor microenvironments. Importantly, our findings align with the study's central thesis that nAChR‐targeted interventions must be carefully tailored to cancer types demonstrating meaningful receptor involvement, as indiscriminate application could lead to suboptimal therapeutic outcomes. This selectivity is further emphasized throughout our discussion, where we address the limitations of generalizing the application of neurotransmitter‐based drugs across heterogeneous cancer populations.

Muscarinic receptors have numerous functions in the human body; for example, modulation of heart rate, vasodilatation, and secretion by glands [45]. Nicotinic receptors, on the other hand, manage the resting potential of neurons, synaptic transmission, and rapid stimulation transmission; thus, they tend to involve cognitive processes: memory, learning, and motor control [46, 47]. Furthermore, studies reveal that nicotinic receptors participate in various diseases ranging from neurological, like Alzheimer's and Parkinson's disease, and epilepsy, to Tourette's syndrome, to psychiatric disorders like schizophrenia, depression, and anxiety [26]. Some missense mutations of the gene coding α4 subunit of nicotinic receptors cause epilepsy [48], while in Alzheimer's and Parkinson's disease, reduced expression of α4β2 and α7 receptors occurs [49, 50]. Activation of nicotinic receptors, on the other hand, increases dopamine release within the striatum in Parkinson's disease [51, 52]. The α4β2 receptor was reported to be involved in anxiety, depression, and addiction with its synergic effects as this receptor modulates mesolimbic dopamine levels [53, 54]. Additionally, α7 nAChRs are implicated in cytokine responses associated with conditions like COVID‐19 [8].

## 4 Expression and Function of nAChRs in Various Cancers

nAChRs are expressed in a range of tissues and tumors in the body (Figure 1). For a long period, it was broadly considered that receptors for neurotransmitters such as nAChRs existed only in the nervous system. Nevertheless, current investigations have revealed a much broader range of operations. Today, such receptors have been discovered to not only be important for neural function but also for regulating non‐neuronal cellular processes, including differentiation, proliferation, and apoptosis. Such processes have been documented in immune cells, lung epithelial cells, and even in cancerous cells, underlining their systemic role [3, 39, 55].

A salient feature of nAChRs is their structural and functional diversity, derived from variation in the arrangement of subunits. For instance, incorporation of the α5 subunit can profoundly increase the sensitivity and responsiveness of receptors and in turn, modulate cellular responses to concentrations of ligands [56, 57]. Notably, even receptors with an equivalent configuration of subunits have been proven to evoke contrasting biological actions about the surrounding cellular environment. As an example, stimulation of an equivalent nAChR subtype in two different cancerous cell types can lead to opposite results, such as proliferation in one case and apoptosis in a contrasting case. These contrasting actions arise through factors such as variation in cell types, progression of cancer, and tissue microenvironments [4, 58].

This complexity is significant for therapies targeting nAChRs. For instance, stimulation of these receptors can inhibit or promote growth, and is dependent on the types of subunits and tissue environment. This reveals that knowing current receptors and their expression levels is crucial but not sufficient for result interpretation, since these factors can undergo alternation in function [4, 39]. Examining these factors in a variety of tissues in future studies will allow us to understand how the contextual dependence of signaling works and will reveal effective targets for therapy. Table 1 provides a concise overview of research findings, illustrating the contextual roles of various nAChR subtypes across different cancer cell lines.

**TABLE 1:** Expression and role of different nAChRs receptors in different cancer types and cell lines.

Receptor subtype	Cancer type	Cell line	Function/attributed pathway		References
α7 nAChR	Breast cancer	MCF‐7	Proliferation and metastasis		[59, 60]
	Prostate cancer	DU145 and PC3	Proliferation and metastasis	EGFR/ERK1/2, PI3K/AKT Pathway	[61, 62]
	Gastric cancer	SGC and HGC cells	Cell survival	PI3K/p‐AKT pathway	[63]
	Lung cancer	NSCLC cell lines	Proliferation, EMT, and drug resistance	β‐arrestin‐Src kinase, PI3K/AKT, and MAPK pathways leading to E2F1 expression, inhibition of binding of the AP2α to the EP4 promoter	[4, 64]
		SCLC cell lines	Proliferation and cell survival	Akt and downstream targets	[65]
α9 nAChR	Breast cancer	MDA‐MB‐231 MCF‐7	Proliferation, metastasis, and cell survival	EGFR/ERK1/2, PI3K/AKT, and MAPK p38 pathways. Involving PKC‐mediated NF‐kB activation	[66, 67]
			Cell survival	Src, and Akt‐mediated upregulation of Bcl‐2	
	Lung cancer	NSCLC cell lines	Cell survival	p44/42 MAPK pathway	[68, 69]
α5 nAChR	Prostate cancer	DU145 and PC3	Proliferation and metastasis	EGFR/ERK1/2, PI3K/AKT pathway	[61, 62]
	Lung cancer	NSCLC cell lines	Proliferation and metastasis	ERK 1/2 and PI3K/AKT and JAK2/STAT3 pathways	[70, 71]
α3‐containing nAChRs	Lung cancer	SCLC cell lines	Proliferation and cell survival	Akt and downstream targets like FKHR, 4‐BEP‐1, P70S6K, and GSK‐3	[72, 73]
α9α10 nAChR	Breast Cancer	MDA‐MB‐157 MCF‐7	Proliferation and inhibition of apoptosis		[74]
α4β2 nAChR	Lung cancer	NSCLC cell lines	Inhibitory role in tumor expansion showing contradictory effects compared to other nAChRs in NSCLC progression		[75, 76]

Abbreviations: EMT, epithelial to mesenchymal transition; NSCLC, non‐small cell lung cancer; SCLC, small cell lung cancer.

The expression and function of receptors in males and females form integral parts that contribute to disease susceptibility and variation in pharmacologic responses. Sex‐dependent variations in the serotonin system are responsible for increased susceptibility to stress and mood disorders in females [77, 78]. Nevertheless, sexual dimorphism in receptor expression continues to be underestimated in pharmaceutical fields [79]. In addition, a difference between males and females in terms of types of receptors for neurotransmitters can produce divergent or similar effects [80]. Sexual variation in regulating neurotransmitters can also impact pathogenesis in cancer, and therapeutic approaches [81, 82]. For example, hormonal, genetic, or epigenetic factors can cause sex‐specific abnormalities in nAChRs in tumors, similar to processes in neuropsychiatric disease.

Investigations have uncovered sex‐related differences in expression for nAChRs, so that females have increased baseline expression of α4β2 nAChRs in most brain regions compared with males. In contrast, long‐term administration of nicotine like smoking due to nicotine addiction induces a larger upregulation of nAChRs in male subjects [83, 84]. For some other types of receptors, significant age and sex‐related differences, in general, are not seen [78]. Thus, it is important to view sex differences in neurotransmitter systems, specifically in regard to nAChRs, in consideration of the acknowledged discrepancies in expression and function between males and females. As emphasized in neuro pharmacologic investigations, ignoring sex differences restricts the development of personalized therapies and veils underlying mechanism understandings [78]. An in‐depth evaluation of sex‐specific expression of nAChRs, signaling networks, and their intersection with cancer‐related networks could reveal new therapeutic targets and/or biomarkers, and therefore, sharpen precision medicine approaches. Consistent with broader calls for expansive research frameworks taking into consideration gender diversity, such an evaluation could promote improvement in diagnostics and therapies in a variety of disease processes.

### 4.1 Breast Cancer

#### 4.1.1 N1(m) nAChR in Breast Cancer

The role of acetylcholine in breast cancer is mediated by its interaction with various receptors, sometimes leading to conflicting effects. Notably, the muscle‐type nicotinic acetylcholine receptor, N1(m) nAChR, has not been extensively studied in breast tumors or tissues other than muscle; thus its impact on breast cancer remains largely unexplored.

The presence of the N1(m) subtype of nAChRs in breast tumors and other non‐muscle tissues has not been extensively documented yet [41, 43], primarily because of the scarcity of available research data on this specific subtype.

Further investigation is needed to elucidate the specific roles of different acetylcholine receptor subtypes, including N1(m) nAChR, in breast cancer development and progression.

#### 4.1.2 N2(n) nAChR in Breast Cancer

Neuronal nAChRs serve an important role in regulating breast cancer tumorigenesis. nAChR subunit expression includes but is not limited to, α3, α4, α5, α7, α9, α10, β2, β3, and β4, and these are found most abundantly expressed in breast cancer cell lines. The greatest attention was placed on α9, which was also significantly higher expressed in breast cancer cell lines [74, 85]. Thus, signaling through the α9 subunit could be the significant trigger of breast carcinogenesis and probably metastasis [86].

**FIGURE 3:**
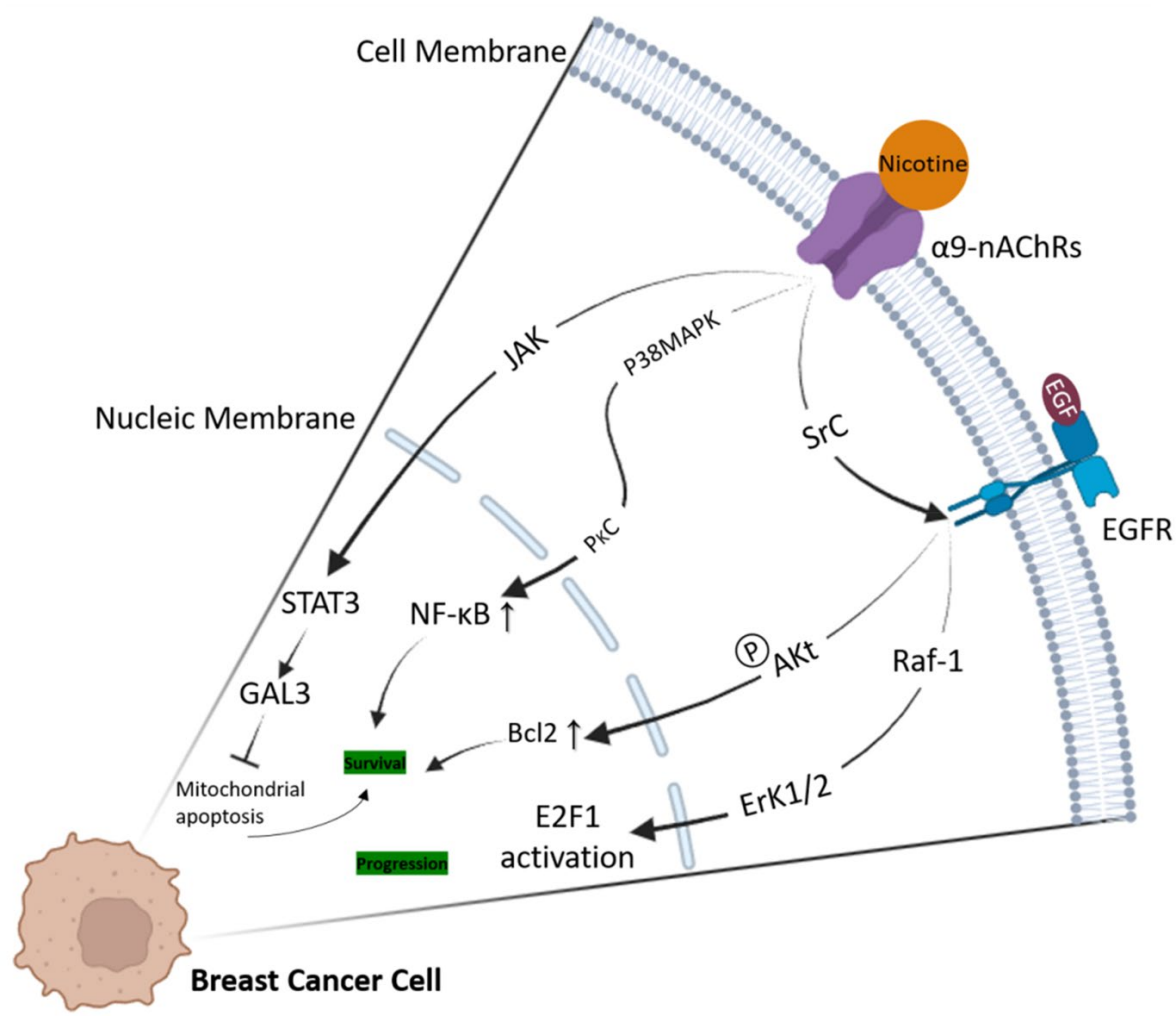
α9‐nAChR signal transduction pathways in breast cancer cells. Various signaling pathways are activated by α9 nAChR in different breast cancer cell lines, contributing to cancer progression. These pathways include activation of the EGFR/ERK1/2 pathway, leading to cell cycle progression via Src, and Akt‐mediated up‐regulation of Bcl‐2 to enhance cell survival. Furthermore, α9‐nAChR activation triggers MAP kinase p38 pathways, involving PKC‐mediated NF‐kB activation, and promotes STAT3 phosphorylation, facilitating its nuclear translocation and increasing transcription of the anti‐apoptotic lectin Gal‐3 gene. Created with BioRender.com.

High levels of α9 expression are associated with advanced stages (III and IV) of breast tumors, and also with increased tumor metastasis and growth, and increased resistance to apoptosis, especially in triple‐negative breast cancer (TNBC) cell lines like MDA‐MB‐231 and MCF‐7 [66, 67]. Apoptosis induced by chemotherapy is largely blunted when α9 is overexpressed [60], and this receptor was effectively targeted in blocking tumor proliferation by Epigallocatechin Gallate (EGCG) [87]. As well, this indicates the significance of this subunit in both homomeric and heteromeric α9‐containing nAChRs like the α9α10 subtype in triggering proliferative and aggressive signals [74]. Activation of α9 nAChR triggers multiple signaling pathways implicated in breast cancer progression, including EGFR/ERK1/2 pathway activation leading to cell cycle progression via Src, and Akt‐mediated upregulation of Bcl‐2 promoting cell survival [88, 89]. Additionally, α9 nAChR activation stimulates PI3K/AKT and MAP kinase p38 pathways, involving PKC‐mediated NF‐kB activation, further promoting cell survival and invasiveness [60, 66, 88]. Research on MCF‐7 breast cancer cells has revealed that increased α9 isoform expression preserves mitochondrial integrity through intracellular Galectin‐3 actions, reducing mitochondrial apoptosis and promoting cell survival. Nicotine stimulation enhances Galectin‐3 expression via STAT3 transcription factor binding, further enhancing apoptosis resistance [90]. Moreover, α9 nAChR activation induces the production of the ABCG2 protein, associated with drug resistance in breast cancer cells. Recent studies have also highlighted the significant impact of α7 nAChRs on breast cancer cell signaling pathways [60]. In normal human breast cell lines (MCF‐10A), α9 nAChR overexpression has demonstrated carcinogenic potential both in vitro and in vivo upon stimulation (Figure 3) [91].

### 4.2 Prostate Cancer

#### 4.2.1 N1(m) nAChR in Prostate Cancer

The role of the muscle‐type nicotinic acetylcholine receptor (N1(m) nAChR) in prostate cancer remains relatively understudied compared with other cancer types. Limited reports suggest that the gene encoding this receptor may be either minimally expressed or inactive in prostate tumor cells [43].

To date, there is sparse evidence indicating significant involvement of N1(m) nAChR in prostate cancer pathophysiology. Further research is required to elucidate the expression patterns and potential functional roles of this receptor subtype in prostate cancer development and progression.

#### 4.2.2 N2(n) nAChR in Prostate Cancer

Some studies have reported neuronal Nicotinic Acetylcholine Receptors' nAChR expression in different components of the urogenital system, including the prostate [61]. In particular, α7nAChR and α5nAChR receptors show high prominence in prostate tissues and are also related to prostate function in their potential impact on prostate cancer development and progression [61, 62].

**FIGURE 4:**
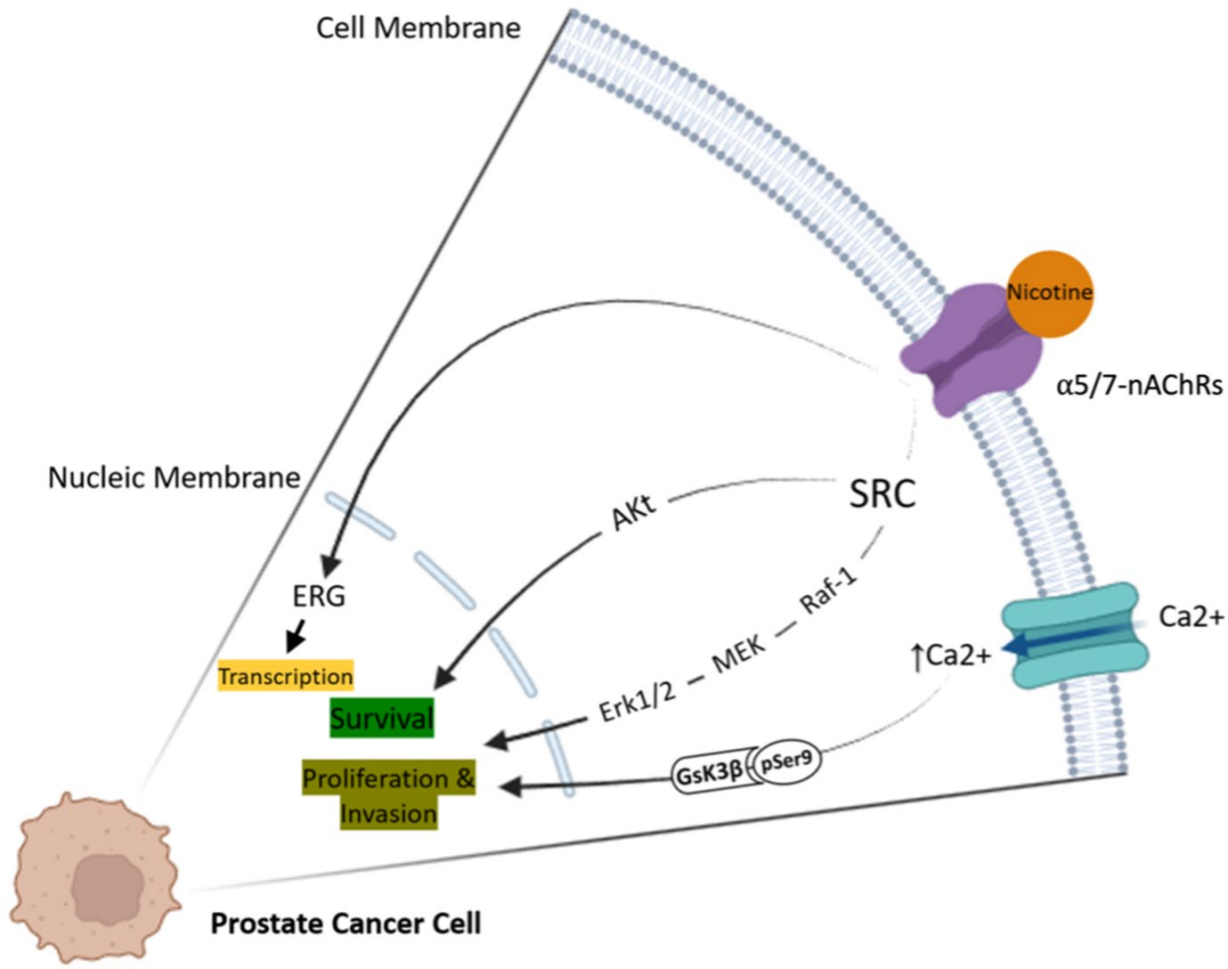
Different nAChRs signal transduction pathways in prostate cancer cells. Activation of nAChR in prostate cancer cells triggers multiple signaling pathways, such as Src‐mediated activation of Akt and Erk1/2 resulting in cell migration, proliferation, and survival. Additionally, it induces increased calcium influx leading to GSK3β(Ser9) phosphorylation, ultimately supporting enhanced proliferation in these cell lines. Created with BioRender.com.

The α5 receptor, being a critical member of the nAChR family, contributes greatly to cancer cell proliferation and invasion in prostate cancer lines, eg, DU145 and PC3. Knockdown of the α5 isoform lowered the phosphorylation of Akt and Erk1/2, alongside reduced cell migration, making this subtype significant in function [61]. Further to this, nicotine dose in LNCap and PC3 showed increased calcium influx and phosphorylation of GSK3β(Ser9), which mediated increased proliferation. It is further noted from clinical cross‐section among smokers with positive TMPRSS2‐ERG gene fusion, calling with clear thrust evidence to prove that nicotine exposure drives prostate tumor progression [92, 93]. In urogenital cancers, including prostate cancer, neuronal nicotinic receptors contribute to tumor development and progression by promoting proliferation, invasion, and metastasis through mechanisms involving cancer stem cells (CSCs). Notably, α5 and α7 subunits are implicated in these processes (Figure 4) [61, 62].

### 4.3 Lung Cancer

#### 4.3.1 N1(m) nAChR in Lung Cancer

Limited reports exist regarding the expression of muscle‐type nicotinic acetylcholine receptor, N1(m) nAChR, in lung cancer tissue. A single study reported high expression of this receptor in non‐small cell lung cancer (NSCLC) cell lines such as 201 and 273 T. Activation of the p44/42 MAPK pathway mediated by N1(m) nAChR was shown to promote cancer cell survival [94]. This finding suggests a potential role for N1(m) nAChR in NSCLC pathophysiology through the modulation of intracellular signaling pathways involved in cell survival. Further research is warranted to better understand the expression patterns and functional significance of N1(m) nAChR in lung cancer, which may have implications for targeted therapeutic approaches.

#### 4.3.2 N2(n) nAChR in Lung Cancer

These neuronal nicotinic acetylcholine receptors are shown to express high levels in different lung cancer cell lines, particularly in NSCLC and small‐cell lung cancer (SCLC) cell lines. Studies have found increased expression of subunits α3, α4, α5, α6, α7, α9, β2, and β4 in NSCLC cell lines [85, 95]. Besides that, most of the studies also found them to be significantly expressed in the following SCLC cell lines for subunits α3, α5, α7, α9, β2, and β4 [96, 97].

The α7 subunit is of particular interest in that it has implicated itself in many progressive roles, such as cell proliferation, repair mechanisms, and tumorigenesis [98, 99]. The significant expression of N2(n) nAChRs in lung cancer cell lines hence demonstrates the prospective importance of these receptors in lung cancer pathophysiology and indicates these receptors as potential therapeutic targets in intervention.

#### 4.3.3 Non‐Small Cell Lung Cancer (NSCLC)

In malignant tumors like NSCLC, the neuronal nAChRs have emerged as important players in the survival and progression of cancer cells based on their expression and function. Best of all, the a9 isoform of nAChRs has been expressed at a high level both in tissues and cell lines derived from NSCLC and is seen as a high activator of p44/42 MAPK pathways thereby increasing cancer survival [68, 69, 94]. Antagonizing α9 nAChR with αBTX or hexamethonium (HEX) inhibits this effect, suggesting a potential therapeutic strategy [100, 101, 102].

The findings demonstrated that activation of α7‐nAChR in the A549 cell lines led to a downstream activation of β‐arrestin which activated the Src kinase and would then cross‐intersect with other mitogenic signaling modules such as PI3 kinase/Akt, Rb‐Raf, and MAPK leading to cancer proliferation in cells [34, 75, 103]. Further, activation of α7‐nAChRs with nicotine also causes further downregulation of epithelial markers, that is, E‐cadherin and β‐catenin, and upregulation of mesenchymal proteins, for example, fibronectin and vimentin, thereby advancing the advantage achieved during the occurrence of epithelial to mesenchymal transition (EMT) in NSCLC cells [34, 104]. Knockdown of α7‐nAChRs impedes nicotine‐induced EMT, thus reaffirming the pivotal role of α7‐nAChRs in NSCLC invasion and metastasis [105]. The activation of the Rb‐Raf‐1/Phospho‐ERK/Phospho‐P90SRK signaling pathway provides a gate through which E2F1 can enter and further contribute to the tumor development and proliferation process in NSCLC [106, 107], while this pathway also activates the antiapoptotic protein mitochondrial Bcl‐2 in lung cancer [68, 108], thereby validating the effective engagement of the α7 subtype for survival in lung cancer.

**FIGURE 5:**
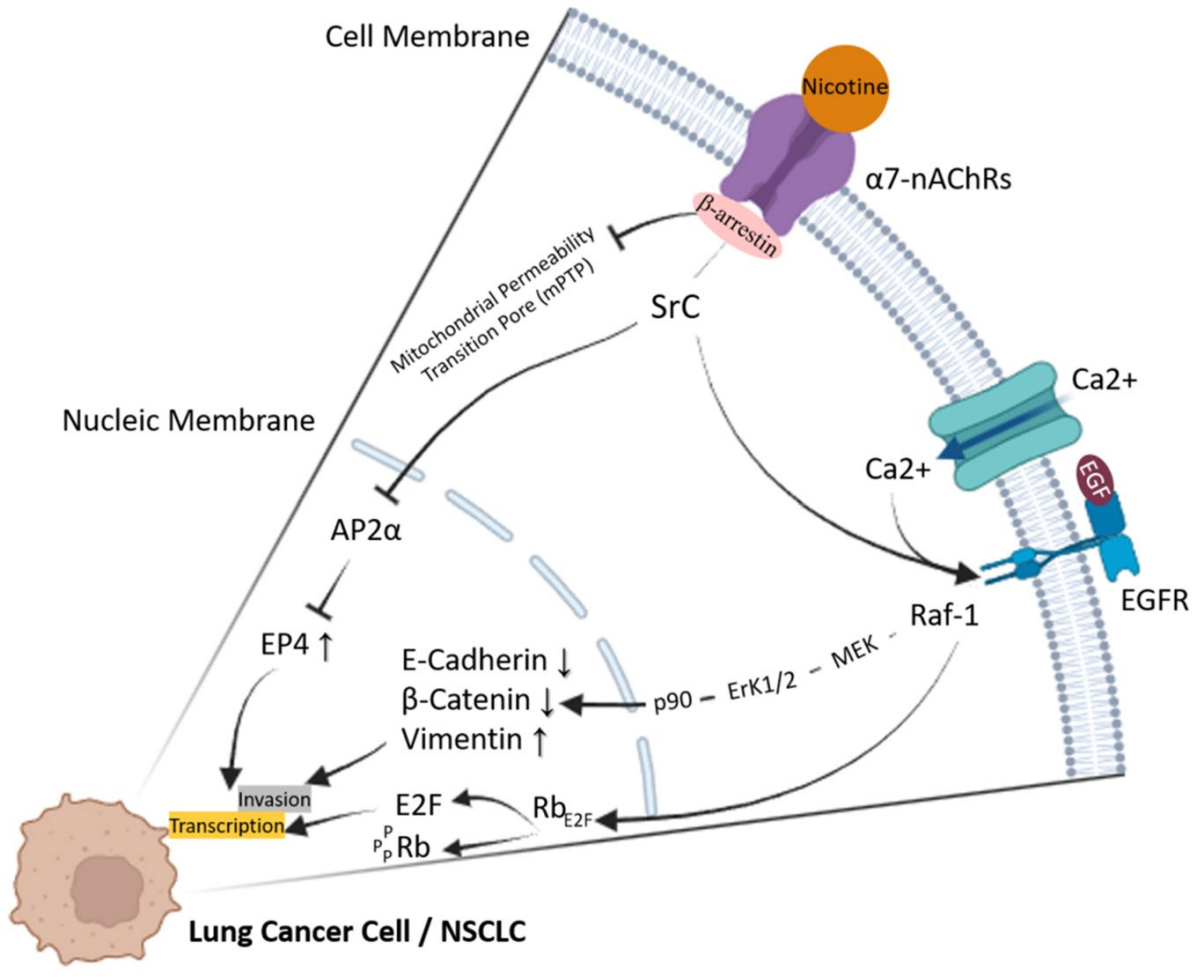
α7‐nAChR signal transduction pathways in NSCLC cells. Activation of α7‐nAChRs triggers β‐arrestin, leading to Src kinase activation and subsequent stimulation of mitogenic signaling pathways such as the Rb‐Raf‐1/Phospho‐ERK/Phospho‐P90SRK signaling pathway, promoting cancer cell proliferation. Furthermore, α7‐nAChR activation leads to the downregulation of epithelial markers (E‐cadherin, β‐catenin) and upregulation of mesenchymal proteins (fibronectin, vimentin), contributing to EMT. Additionally, α7‐nAChR activation inhibits the binding of AP2α to the EP4 promoter, resulting in enhanced cell proliferation. Created with BioRender.com.

α5‐nAChR is also overexpressed in NSCLC. This subtype is responsible for the proliferation, migration, and invasion of tumor cells by the direct activation of ERK 1/2 and PI3K/AKT signaling pathways [61, 68, 109]. Some studies propose that α5‐nAChR mediates nicotine‐induced lung cancer through activation of the JAK2/STAT3 signaling pathway, establishing the mutual interaction between α5‐nAChR and STAT3. Disruption of α5‐nAChR could represent hence one of the new strategies that could hinder nicotine‐promoted lung cancer cell proliferation. This study confirms that the same signaling pathway is activated, even though the triggering receptor may differ. Moreover, α5 and α7 isoforms are upregulated in NSCLC and correlate with poor prognosis and decreased patient survival. Conversely, α4β2 nAChRs exhibit an inhibitory role in tumor expansion, indicating complex and contradictory effects of nicotinic receptors in NSCLC progression (Figure 5) [75, 76, 110].

#### 4.3.4 Small Cell Lung Cancer (SCLC)

In SCLC, nicotine exposure has been shown to promote cell survival and proliferation in various cell lines. In the DMS‐53 line, the expression of α3, α4, and α5 variants was found to correlate significantly with lung cancer tissue sensitivity, highlighting the importance of these genes for cancer cell survival. Notably, selective agonists like α‐Conotoxin Aulb targeting α3β4‐nAChRs effectively inhibit the survival of SCLC cells [41].

**FIGURE 6:**
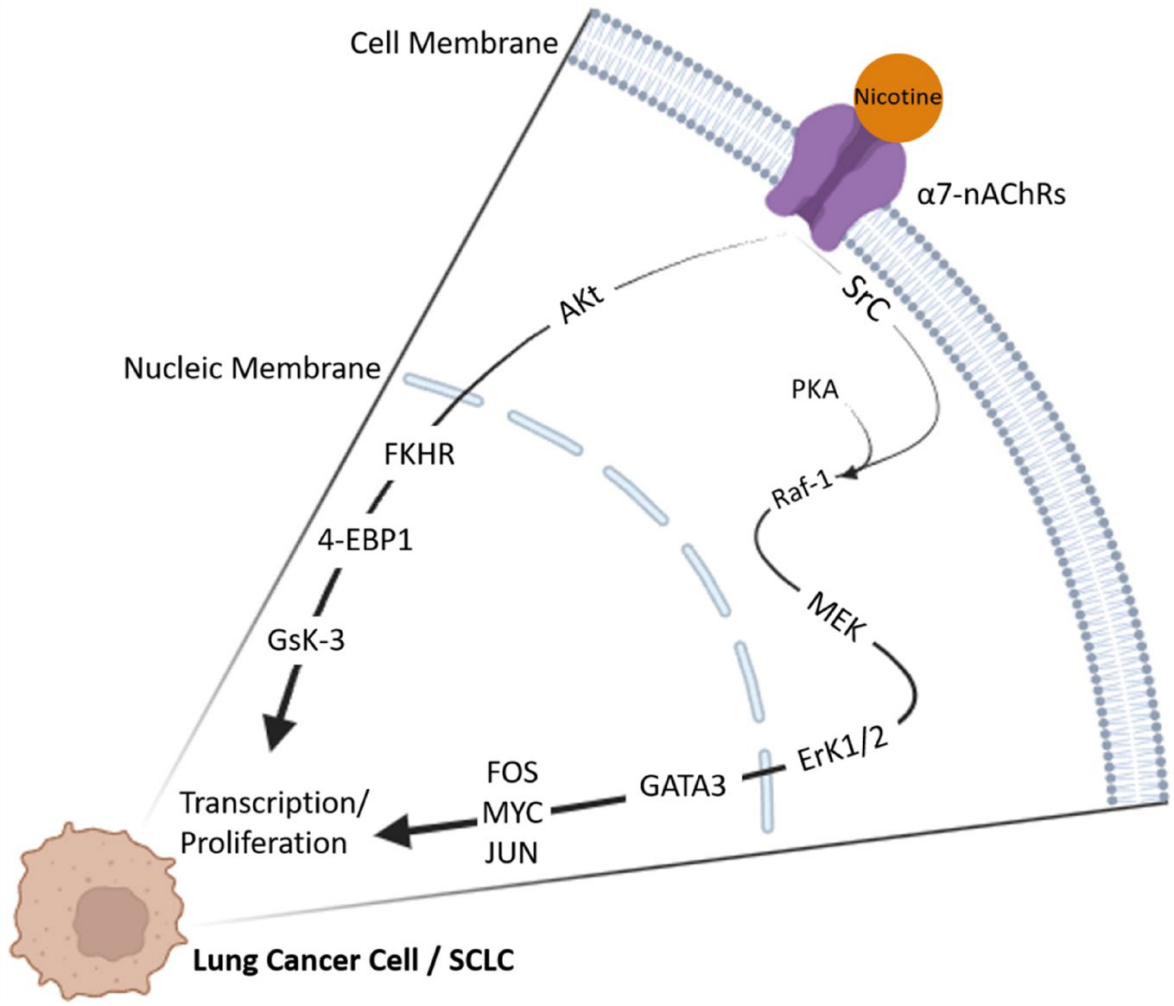
Stimulation of α7‐nAChR in SCLC cells triggers the activation of essential proteins such as Raf‐1, ERK1, and ERK2, as well as transcription factors FOS, JUN, and MYC. Nicotine exposure also results in the activation of Akt and subsequent phosphorylation of downstream targets like FKHR, 4‐BEP‐1, P70S6K, and GSK‐3, promoting cancer cell progression. Created with BioRender.com.

Nicotine compounds and derivatives such as *nicotine‐derived nitrosamine ketone* (NNK) 4‐(methylnitrosamino)‐1‐(3‐pyridyl)‐1‐butanone and N nitrosonornicotine impact α7 receptor expression in the BEP2D line, increasing proliferative activity [68, 69, 111]. NNK and N nitrosonornicotine activate key proteins and kinases including STAT1, NF‐κB, GATA3, protein kinase A, Raf‐1, ERK1, ERK2, and transcription factors FOS, JUN, and MYC in SCLC [62, 75, 112]. Nicotine‐induced activation of Akt and downstream phosphorylation of components like FKHR, 4‐BEP‐1, P70S6K, and GSK‐3 involves α3, α4, and α7 receptors [72, 113]. Genetic variations in the CHRNA3/5 gene, responsible for coding α3 and α5 nAChRs subunits, are strongly linked to lung cancer development [114]. Activation of these receptors by nicotine not only promotes cancer cell growth but also affects the tumor microenvironment, impacting neighboring stroma (Figure 6) [39, 69, 115].

## 5 Discussion

In analyzing the initial data exploring the potential links between psychological states and tumor status, several key insights emerge. Our study focused on neurotransmitters like acetylcholine, positing their significant role in shaping individuals' moods and emotions. However, it's essential to recognize that additional signaling pathways beyond neurotransmitters may influence psychological well‐being. Exploring hormonal pathways, such as oxytocin and endorphins, could provide valuable insights into the complex interplay of chemical processes regulating emotions [116, 117, 118]. This raises intriguing possibilities about other substances that may modulate the intricate connection between the psyche and bodily functions, warranting further investigation [119].

Here in this review, for the purpose of getting a specific conclusion regarding the modulators of different neurotransmissions and impartial decisions, we have considered nicotinic acetylcholine receptors only. Meanwhile, there were reported common responses of other receptors, i.e., muscarinic receptors, like triggering the MAPK/ERK and PI3K/Akt pathways by releasing Ca + 2 [3, 120]. This refers to the triggering of common pathways even with diverse receptors. More studies must give a holistic picture of pathways involving not just acetylcholine but also serotonin and dopamine, to be sure of the presence of efficient common pathways. The interplay between signaling pathways and different forms of cancer remains a largely unexplored area, emphasizing the need for comprehensive research. We also question the validity of the psychological criteria underpinning statistical data and advocate for more interdisciplinary approaches to unravel these intricate relationships.

## 6 Conclusion

This study offers a comprehensive exploration of the influence of activating nAChRs in breast, prostate, and lung cancer, emphasizing the underlying signaling pathways. Drawing from evidence linking emotions to cancer development and regulation, we hypothesized that the signaling pathways implicated in psychiatric outcomes also contribute significantly to tumorigenesis. Although this study exclusively focused on nAChR‐activated pathways, further research is essential to fully comprehend the interplay between neurotransmitter levels in the CNS and the PNS, a topic only briefly touched upon here [26, 27].

Lung cancer progression is notably fueled by the activity of the α7 nAChR subunit, which plays a key role in promoting cancer cell growth and survival. By activating Src kinase, ERK1/2, NF‐κB, and various STAT pathways, the α7 subunit transmits powerful signals that contribute to lung cancer progression [68, 69]. Similarly, in breast cancer, the α9 nAChR subunit is pivotal in driving cell growth and survival through similar signaling pathways like Src kinase, advancing breast cancer development [60]. Further investigation is required to fully understand the roles of specific nAChR subunits in promoting cell growth and survival in prostate cancer. While preliminary evidence suggests the α5 and α7 subunit's significant impact on prostate cancer leading to the common pathways mentioned above, comprehensive studies are necessary to fully elucidate its function [61, 62].

Discrepancies observed across studies may stem from receptors eliciting varied responses in different cancer cell groups. Additionally, receptor expression levels are crucial in determining their specific effects [121]. Notably, the absence of reports on N1(m) receptor expression in these cancers underscores the importance of exploring receptor‐specific signaling effects. Tumor cells can undergo functional alterations, leading to unpredictable responses, highlighting the need for meticulous research to confirm or challenge existing hypotheses despite encountering contradictions [122].

## 7 Clinical Implications

The study's findings suggest the need for caution when it comes to the evaluation and use of the aforementioned substances regarding neurochemical medications for patients suffering from cancer, especially in consideration of the type of cancer to be sure that interaction with pathways for tumor growth possibility is avoided. However, it is now a matter of course for a patient undergoing treatment for cancer with certain pharmaceuticals targeting neurotransmitter systems, such as antidepressants and antipsychotics, to be prescribed those drugs [123, 124]. However, caution is warranted due to the complexity of interactions. For instance, while reports highlight the potential cytotoxic effects of elevated serotonin levels in tumors [125], serotonin has also been shown to exhibit synergistic effects on tumor growth [126]. Therefore, a nuanced consideration of cancer type before administering antidepressants is crucial. Similarly, dopamine agonist drugs have shown promise in inhibiting tumor growth in specific cancers by activating the receptor [127], while receptor antagonists like thioridazine may be recommended to suppress tumor growth in other cases [128]. In the case of acetylcholine, agonists like Carbachol are generally discouraged in cancer patients due to their potential to exacerbate tumor growth [129], whereas recent research has identified several nAChRs antagonists that show significant potential in targeting specific cancer types, providing promising avenues for treatment.

In the case of breast cancer, antagonists for α9‐containing nAChRs have proven to be effective in a significant manner. In specific, α‐Conotoxins have proven to effectively inhibit metastasis, tumor growth, and migration of cells [41, 130]. For instance, αO‐Conotoxin GeXIVA, a potent antagonist for the α9α10 nAChR subtype, inhibits proliferation in MDA‐MB‐157 breast cancer cells through down‐regulation of this nAChR and induces cell cycle arrest [74, 85]. In addition, administration of α‐Bungarotoxin as a selective antagonist for the α7 subunit of nAChRs effectively treated breast tumors. All these observations suggest that nicotine can promote the population of stem cells through α7‐nAChR and PKC‐Notch‐dependent signaling in MCF‐7 cells [89, 131].

For NSCLC, α7‐nAChR antagonists have proven to have significant therapeutic activity. Agents such as β‐alkylpyridinium polymers (Poly‐APS) have been seen to induce apoptosis via mitochondrial routes [132, 133], and QND7 works through inhibition of the Akt/mTOR survival and progression‐related cascade in tumors [99]. D‐tubocurarine and α‐cobratoxin (α‐CbT) have the potential to inhibit factors in tumor cells that promote growth in reaction to nicotine stimulation, and in doing so, make the tumor sensitive to reduced chemotherapy dosages [134, 135]. Notably, β‐Cryptoxanthin inhibits migration and invasion of α7 nAChR‐expressing NSCLC cells through down‐regulation of the AKT/PI3K cascade [69, 136]. On the other hand, in SCLC, small‐molecule antagonist MG624 showed promise through inhibition of angiogenesis and proliferation of tumors through α7 nAChRs, and subsequently, suppression of nicotine‐evoked fibroblast growth factor 2 (FGF2) [137, 138]. In addition, α‐bungarotoxin (α‐BTx), a selective α7 nAChR antagonist, disrupts metastatic progression in SCLC and NSCLC through inhibition of phosphorylation routes involved in metastasis, namely through proto‐oncogene tyrosine‐protein kinase Src and additional kinases about stimulation with *NNK* [65, 139, 140]. However, despite robust shared evidence regarding the role of nicotinic acetylcholine receptor signaling pathways in prostate cancer, further research is still required to investigate the effects of various compounds on prostate tumors.

Therefore, in certain cases, stimulation of nAChRs, selectively targets malignant cells with little impact on surrounding healthy tissue, offering a therapeutic path with a positive prognosis. According to studies, administration of nAChR antagonists in combination with conventional chemotherapy protocols forms a more effective therapeutic intervention through increased susceptibility of tumors to lowered dosages of chemotherapeutic drugs and reduced toxicity with high dosages. In cases of overexpressed nAChRs in tumors, such a therapeutic intervention can prove particularly beneficial [141, 142]. This nuanced examination could lead to more customized treatment methods and improved outcomes for patients. Moreover, the identification of specific tumor signaling pathways associated with nicotinic acetylcholine receptors introduces new possibilities for targeted treatments across various types of cancer. Healthcare providers may modulate these pathways to achieve patient welfare enhancement and potential mortality rate reductions in the future. Including interdisciplinary approaches that offer psychological and biological perspectives could, however, open up even better possible treatment avenues for cancer over an even more complex interplay between tumor development and emotion.

## 8 Study Limitations

Despite the valuable insights provided by the study, several limitations must be acknowledged. The intricate connections between neurotransmitters, emotions, and tumor growth present a significant challenge in fully understanding these mechanisms, potentially limiting the generalizability of the findings [26, 33]. Furthermore, concerns arise regarding the reliability of psychological criteria used in statistical data, raising questions about the accuracy of diagnosing depression in cancer patients and underscoring the necessity for enhanced assessment methods [143, 144]. The study underscores the underexplored interplay between signaling pathways and different cancer types, revealing gaps in current understanding that warrant further investigation. It advises caution in administering neurotransmitter‐based medications to cancer patients, given their potential negative impact on tumor growth. This highlights the critical importance of carefully evaluating the risks and benefits of such treatments [125].

## Author Contributions

**Mohammad Hossein Azadi** conceptualization and design of the study, drafting figure schematics, acquisition of references, and critical review and editing. **Pouya Pazooki:** manuscript revisions, figure generation, critical reviewing, and approving the final version of the manuscript. **Soheila Ajdary:** manuscript revisions, critical review, and editing. **Hamed Shafaroodi:** conceptualization and design of the study, manuscript revisions, and approving the final version of the manuscript. All authors have read and approved the final manuscript.

## Conflicts of Interest

The authors declare no conflicts of interest.

## Data Availability

All the data is available upon contacting the corresponding author.
